# Matrix-associated autologous chondrocyte implantation with autologous bone grafting of osteochondral lesions of the talus in adolescents: patient-reported outcomes with a median follow-up of 6 years

**DOI:** 10.1186/s13018-021-02384-8

**Published:** 2021-04-08

**Authors:** Daniel Körner, Christoph E. Gonser, Stefan Döbele, Christian Konrads, Fabian Springer, Gabriel Keller

**Affiliations:** 1 Department of Traumatology and Reconstructive Surgery, BG Trauma Center Tübingen, Eberhard Karls University Tübingen, Schnarrenbergstr. 95, 72076 Tübingen, Germany; 2 Department of Diagnostic and Interventional Radiology, University Hospital Tübingen, Eberhard Karls University Tübingen, Hoppe-Seyler-Str. 3, 72076 Tübingen, Germany; 3 Department of Radiology, BG Trauma Center Tübingen, Eberhard Karls University Tübingen, Schnarrenbergstr. 95, 72076 Tübingen, Germany

**Keywords:** Osteochondral lesion, Ankle, Talus, Children, Adolescents, Autologous bone grafting, Autologous chondrocyte implantation, Matrix-associated autologous chondrocyte implantation, Patient-reported outcome measures, Osteoarthritis

## Abstract

**Background:**

This study presents patient-reported outcome measures after combined matrix-associated autologous chondrocyte implantation and autologous bone grafting in high-stage osteochondral lesions of the talus in adolescents.

**Methods:**

A total of 12 adolescent patients (13 ankles) received matrix-associated autologous chondrocyte implantation and autologous bone grafting for a solitary osteochondral lesion of the talus at a single centre. The Foot and Ankle Outcome Score and Foot and Ankle Ability Measure were defined as outcome measures (median follow-up 80 months [range 22–107 months]). Pre- and postoperative ankle radiographs were evaluated according to the van Dijk ankle osteoarthritis scale.

**Results:**

The study population consisted of four male and nine female cases (mean age at the time of surgery, 17.7 ± 2.1 years). Eight lesions were classified as traumatic and five as idiopathic*.* Twelve lesions were located medial vs one lateral in the coronal plane and all central in the sagittal plane. The median lesion size and depth were 1.3 cm^2^ (range 0.9–3.2 cm^2^) and 5 mm (range 5–9 mm), respectively.

There were no perioperative complications in any of the cases.

In 9 cases patient-reported outcome measures were available. The results of the Foot and Ankle Outcome Score subscales were *symptoms*, 70 ± 14; *pain*, 83 ± 10; *activities of daily living*, 89 ± 12; *sports/recreational activities*, 66 ± 26; and *quality of life*, 51 ± 17. The mean overall Foot and Ankle Outcome Score was 78 ± 13.

The results of the Foot and Ankle Ability Measure subscales were *activities of daily living*, 81 ± 20; *function/activities of daily living*, 84 ± 13; *sports*, 65 ± 29; and *function/sports*, 73 ± 27. According to the *function overall* subscale of the Foot and Ankle Ability Measure, in two cases, the patients assessed the ankle function as normal, in three as nearly normal, and in three as abnormal (missing data, *n* = 1).

Preoperative van Dijk scale: stage 0 in five cases and stage I in eight cases; postoperative van Dijk scale: stage 0 in four cases, stage I in 9 cases

**Conclusions:**

Patient-reported outcome measures following matrix-associated autologous chondrocyte implantation and autologous bone grafting for high-stage osteochondral lesions of the talus in adolescents show heterogeneous results. Long-term limitations mainly affect sports and recreational activities. Osteochondral lesions of the talus are associated with osteoarthritis, even preoperatively. However, we did not find significant osteoarthritis progression after matrix-associated autologous chondrocyte implantation and autologous bone grafting in the long term.

## Background

Osteochondral lesions of the talus (OCLTs) are rare in pre-school children but become more frequent in schoolchildren and especially female adolescents [1]. Except for acute flake fractures, the primary treatment is conservative [2]. Operative therapy options of OCLTs in the paediatric and adolescent population vary; there is a lack of internationally accepted treatment guidelines. For low-stage OCLTs, good clinical results have been reported after arthroscopic treatment with bone marrow stimulation (BMS) techniques, fragment fixation, and retrograde drilling [3–8]. However, relatively high re-operation rates due to treatment failure have also been reported [9]. There is little evidence regarding the operative treatment of high-stage OCLTs in children and adolescents.

Different expert groups have proposed thresholds for cartilage replacement therapies such as matrix-associated autologous chondrocyte implantation (MACI) and matrix-induced bone marrow stimulation (M-BMS) at a defect size of 1.0 cm^2^ [10] and 1.5 cm^2^ [11, 12], respectively. In the case of deep subchondral bone loss, additional autologous bone grafting (ABG) can be performed. The threshold for bone grafting in addition to scaffold-based therapies (MACI or M-BMS) has been defined at a defect depth of 3 mm [10] and 5 mm [12], respectively.

In a large case series, Kramer et al. analysed the results of surgical treatment of 109 osteochondral lesions of the ankle (107 OCLTs and two osteochondral lesions of the distal tibia) of 100 patients with a mean age of 14.3 ± 2.3 years over a median follow-up period of 3.3 years [9]. The patient population showed Berndt/Harty stages I to IV lesions. Different surgical techniques such as transarticular drilling (54%), fragment fixation (20%), and excision microfracture (26%) were used. The authors found that female sex and higher BMI were associated with worse Foot and Ankle Outcome Score (FAOS) outcomes. However, no significant difference in FAOS scores was detected according to the procedures used or the postoperative radiographic findings [9].

To the best of our knowledge, no study has yet reported long-term clinical outcome data after MACI/ABG in a paediatric and adolescent patient population with high-stage OCLTs. The aim of this study is to present patient-reported outcome measures (PROMs) after combined MACI/ABG in adolescents.

## Methods

### Study population

Between 2012 and 2018, 12 adolescent patients (13 ankles) received MACI/ABG for a solitary OCLT at our trauma centre. The patient files and imaging data that were digitally available in the picture archiving and communication system were retrospectively analysed. Further, the patients were followed up using PROMs. The following inclusion criteria were defined: presence of a solitary OCLT, operative treatment with MACI/ABG at our institution, and patient age 20 years or younger at the time of MACI/ABG. The World Health Organization defines adolescence as the period of life between the ages of 10 and 20.

### Data capture

Epidemiological data such as age at the time of MACI/ABG, gender, and body mass index (BMI) were captured. Furthermore, data about the OCLT were analysed, such as the side of injury and the location at the talus (coronal: medial vs central vs lateral; sagittal: anterior vs central vs posterior). It was recorded whether the tibial plafond showed a corresponding lesion.

The aetiology of the OCLT was classified as traumatic when there was a history of trauma in the medical history of the patient. Otherwise, the OCLT was classified as idiopathic.

The OCLTs were classified according to the International Cartilage Repair Society (ICRS) classification and the Berndt-Harty-Loomer (BHL) classification [13]. The lesion size and depth were evaluated based on preoperative cross-sectional imaging (preoperative magnetic resonance imaging was available for retrospective analysis in eight cases, computed tomography in eleven cases). The lesion size was calculated by multiplying length and width.

It was registered whether and when there had been previous operations to that ankle/OCLT. Complications following MACI/ABG were also captured.

### Operative technique

The decision for MACI/ABG was based on medical history, clinical examination findings, preoperative imaging, patient preferences, and findings in ankle arthroscopy. MACI/ABG was considered in the case of ICRS stage 4 cartilage damage with a lesion size ≥ 1.0 cm^2^ and/or lesion depth ≥ 5 mm.

MACI/ABG was performed as a two-stage procedure. In the first step, ankle arthroscopy was performed to evaluate the articular cartilage. If the indication for MACI/ABG was confirmed, cartilage was harvested from an area outside the main weight-bearing zone of the talus and sent to the manufacturer. In one case, arthroscopy with retrograde drilling of the OCLT was combined with chondrocyte harvesting.

After cultivation, the MACI scaffold was implanted in a second procedure. Novocart® 3D (Tetec AG, Reutlingen, Germany) was used in nine cases, and Novocart® Inject (Tetec AG, Reutlingen, Germany) was used in four cases. After medial (*n* = 12) or lateral (*n* = 1) malleolar osteotomy, the OCLT was debrided, and sharp cartilage rims were cut. For ABG, bone cylinders were harvested from the ipsilateral (*n* = 11) or contralateral (*n* = 2) iliac crest and placed into the bony talar defect. The MACI scaffold was placed onto the bone. In the case of Novocart® 3D, the matrix was fixed to the surrounding cartilage with absorbable sutures. The osteotomy of the malleolus was fixed with tension band wiring (*n* = 5), screw fixation (*n* = 7), or plating (*n* = 1, lateral malleolus).

The rehabilitation protocol consisted of partial weight bearing (20 kg) in a lower leg orthosis for 6 weeks after the procedure.

In seven cases, hardware removal was performed in our institution combined with follow-up arthroscopy (mean time between MACI/ABG and hardware removal/follow-up arthroscopy 12 ± 5 months). One of those patients underwent a revision operation in the further course because of cyst formation at the medial malleolus (case no. 3). In the remaining cases, the hardware removal was performed without arthroscopy (*n* = 3) or in another hospital (*n* = 3).

### Outcome measures

The German versions of the FAOS [14] and Foot and Ankle Ability Measure (FAAM) [15] were defined as outcome measures. The questionnaires were sent to the patients; they were asked whether they had undergone any re-operation after treatment at our institution. Eight of the 12 patients with nine MACI/ABG procedures returned the completed PROMs. The median follow-up was 80 months (range 22–107 months).

Furthermore, both preoperative and the last available postoperative ankle radiographs (anteroposterior and lateral views) were evaluated according to the van Dijk ankle osteoarthritis (OA) scale (median follow-up 8 months [range 1–67 months]) [16, 17].

### Statistical analysis

Statistical analysis was performed using the software package JMP (SAS Institute Inc., JMP, Version 12.2.0, Cary, NC, USA). The Shapiro-Wilk *W* test was applied to screen the data for normality of distribution. Mean and standard deviation for normally distributed data as well as median and range for non-normally distributed data were reported. Descriptive statistics were carried out.

### Compliance with ethical standards

The study was performed according to the ethical standards of the institutional and national research committee and according to the 1964 Helsinki Declaration and its later amendments. The institutional review board approved this study (identification number: 180/2020BO1).

Patients who returned the PROMs gave their informed consent. For patients who were lost to follow-up (*n* = 4), consent was waived, since in these patients only a retrospective, pseudonymised data evaluation was performed.

The authors declare that they have no potential conflicts of interest.

## Results

Table 1 shows the demographic and lesion characteristics of the study population. The study population consisted of four male and nine female cases in 12 patients (mean age at the time of MACI/ABG 17.7 ± 2.1 years). The median BMI was 23.5 kg/m^2^ (range 18.0–39.3 kg/m^2^). The distal tibial physis was closed in all patients. The median lesion size and depth were 1.3 cm^2^ (range 0.9–3.2 cm^2^) and 5 mm (range 5–9 mm), respectively.

**Table 1:** Demographic and lesion characteristics

Patient	Case	Age (years)	Sex	BMI (kg/m^2^)	Aetiology	Previous operations	Side	Location		Tibial lesion	ICRS	BHL	Size (cm^2^)	Depth (mm)
								Coronal	Sagittal					
1	1	17	F	24.6	T	2: Arthroscopy, retrograde drilling^a^; re-arthroscopy, retrograde drilling, BMS distal tibia	R	Med.	C.	Yes	4	3	1.4	5
1	2	19	F	25.4	T	0	L	Med.	C.	No	4	3	1.0	5
2	3	18	F	23.3	I	0	R	Med.	C.	No	4	3	1.1	5
3	4	14	F	18.1	I	0	R	Med.	C.	No	4	3	^b^	^b^
4	5	20	F	18.0	T	0	R	Lat.	C.	No	4	4	1.2	5
5	6	20	M	23.5	I	1: Arthroscopy, retrograde drilling	R	Med.	C.	No	4	4	1.1	9
6	7	20	F	18.8	I	0	L	Med.	C.	No	4	3	1.1	5
7	8	15	F	24.0	T	2: Arthroscopy^a^; re-arthroscopy, BMS	R	Med.	C.	No	4	3	1.9	7
8	9	20	M	26.0	T	0	L	Med.	C.	No	4	3	0.9	5
9	10	15	F	30.5	I	0	L	Med.	C.	No	4	3	1.6	5
10	11	18	M	23.5	T	0	L	Med.	C.	No	4	4	3.2	5
11	12	16	F	22.3	T	1: Arthroscopy, retrograde drilling	R	Med.	C.	No	4	3	1.6	5
12	13	18	M	39.3	T	0	R	Med.	C.	Yes	4	3	1.6	6

*BHL*, Berndt-Harty-Loomer classification; *BMI*, body mass index; *BMS*, bone marrow stimulation; *ICRS*, International Cartilage Repair Society classification; *F*, female; *M*, male; *I*, idiopathic; *T*, traumatic; *L*, left; *R*, right; *Lat*., lateral; *Med*., medial; *C*., central

^**a**^Other hospital

^**b**^Missing data

There were no perioperative complications in any of the cases after MACI/ABG (*n* = 13). In one case (case number 3), there were complications during hardware removal/follow-up arthroscopy 11 months after MACI/ABG, with difficult screw removal from the medial malleolus. In the further course, an osseous cyst developed on the medial malleolus, which was finally treated with autologous bone grafting after 14 months. The remaining cases in the follow-up population had no re-operation within the follow-up period. The cases lost to follow-up (*n* = 4) had no re-operations for the period when they were treated in our institution (median 7 months, range 1–13 months).

**Fig. 1:**
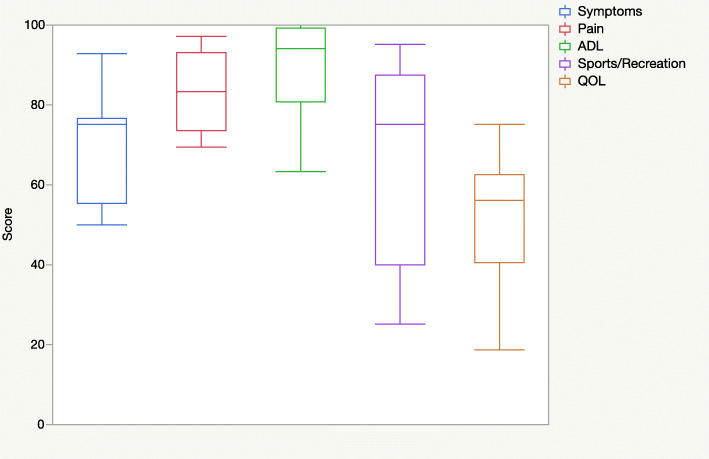
Box plots of the five Foot and Ankle Outcome Score subscales (mean ± standard deviation): symptoms, 70 ± 14; pain, 83 ± 10; activities of daily living (ADL), 89 ± 12; sports/recreational activities, 66 ± 26; quality of life (QOL), 51 ± 17

**Fig. 2:**
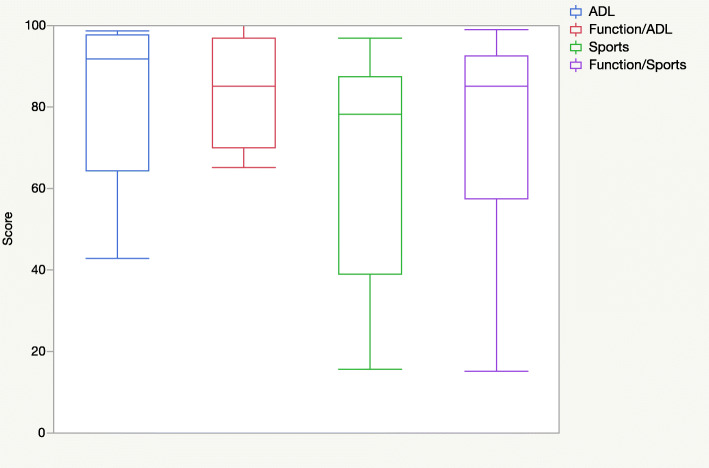
Box plots of the four Foot and Ankle Ability Measure subscales (mean ± standard deviation): activities of daily living (ADL), 81 ± 20; function/ADL, 84 ± 13; sports, 65 ± 29; function/sports, 73 ± 27

The clinical outcomes according to the FAOS and FAAM scores for every case are presented in Table 2. The mean overall FAOS score was 78 ± 13. Figure 1 shows the results of the five FAOS subscales, and Fig. 2 shows the results of the FAAM subscales *activities of daily living*, *function/activities of daily living*, *sports*, and *function/sports*.

**Table 2:** Patient-reported outcome scores (Foot and Ankle Outcome Score and Foot and Ankle Ability Measure)

Patient	Case	FU (months)	Foot and Ankle Outcome Score						Foot and Ankle Ability Measure				
			*Symptoms*	*Pain*	*ADL*	*Sports/recreation*	*QOL*	*Overall*	*ADL*	*Function/ADL*	*Sports*	*Function/sports*	*Function overall*
1	1	107	75	83	94	80	56	83	94	90	78	90	Nearly normal
1	2	83	93	97	100	95	69	95	98	95	91	95	Normal
2	3	96	75	78	85	60	44	75	61	80	47	70	Missing
3	4	L											
4	5	96	61	69	63	25	38	57	43	65	16	15	Abnormal
5	6	80	50	69	76	40	19	61	70	75	31	45	Abnormal
6	7	L											
7	8	47	71	97	100	80	75	90	99	100	84	85	Nearly normal
8	9	L											
9	10	L											
10	11	26	75	83	94	75	56	83	92	85	84	70	Nearly normal
11	12	24	50	78	90	40	44	70	77	65	53	85	Abnormal
12	13	22	79	89	99	95	56	89	98	99	97	99	Normal

*FU*, follow-up; *ADL*, activities of daily living; *QOL*, quality of life; *L*, lost to follow-up

Table 3 indicates the pre- and postoperative radiographic findings according to the van Dijk ankle OA scale. In eight cases (62%), the preoperative radiographs showed stage 1 OA (vs five cases [38%] without signs of OA). In the postoperative radiographs, nine patients (69%) had stage 1 OA and four patients (31%) stage 0 OA.

**Table 3:** Radiographic outcome (van Dijk scale)

Patient	Case	Pre	Post	FU (months)
1	1	1	1	67
1	2	0	1	43
2	3	1	1	11
3	4	0	0	8
4	5	0	0	12
5	6	1	1	1
6	7	1	1	6
7	8	1	1	17
8	9	1	1	1
9	10	1	1	4
10	11	1	1	23
11	12	0	0	2
12	13	0	0	2

Van Dijk scale before and after matrix-associated autologous chondrocyte implantation and autologous bone grafting (MACI/ABG). The latest radiograph before the operation “pre” and the latest available radiographs “post” were used, respectively. In case 3, the last radiograph prior to the revision operation at the medial malleolus was used as the latest follow-up radiograph to minimise bias. The time between MACI/ABG and postoperative radiograph was defined as the follow-up (FU)

Patients who assessed their *function overall* according to the FAAM score to be normal (*n* = 2) or nearly normal (*n* = 3) showed stage 1 OA in four cases (80%) and no signs of OA in one case (20%) on postoperative radiographs. In contrast, in patients who assessed their *function overall* to be abnormal (*n* = 3), postoperative radiographs showed stage 1 OA in one case (33%) and no signs of OA in two cases (67%).

Cases without previous operation regarding the affected ankle (*n* = 9) had a mean age of 18.0 ± 2.2 years at the time of MACI/ABG. The group consisted of three males and nine females (33% vs 67%), and the median BMI was 23.5 kg/m^2^ (range 18.0–39.3 kg/m^2^). In contrast, the group of cases with one or two previous operations (*n* = 4) had a mean age of 17.0 ± 2.2 years, a gender distribution of one male vs three females (25% vs 75%), and a median BMI of 23.75 kg/m^2^ (range 22.3–24.6 kg/m^2^).

Cases without previous operation showed no signs of OA in four cases (44%) and stage 1 OA according to the van Dijk classification in five cases (56%) on pre-MACI/ABG radiographs compared to cases with previous operation, which showed no signs of OA in one case (25%) and stage 1 OA in three cases (75%) prior to MACI/ABG.

## Discussion

The aim of this study is to present clinical results after MACI/ABG for OCLTs in adolescents using PROMs. PROMs are increasingly used to evaluate the subjective outcomes of surgical interventions of the foot and ankle [18, 19]. An analysis of the measurement properties of the FAOS and FAAM scores identified these as promising PROMs [19].

The comparability of FAOS and FAAM score values between different patients is limited as the scores represent a highly subjective patient point of view. The subjective outcomes after MACI/ABG in this study were heterogeneous. In some subscales, the values were widely scattered between the patients (e.g., FAOS subscale *sports/recreational activities*, FAAM subscales *sports* and *function/sports*). Overall, some patients seem to have long-term limitations, especially in sport activities (as seen in the FAOS subscale *sports/recreational activities* and the FAAM subscales *sports* and *function/sports*). In addition, quality of life seems to be impaired in the long term in some patients (FAOS subscale *QOL*).

When interpreting the PROMs, it must be taken into account that only high-stage OCLTs (stage 4 cartilage damage according to the ICRS classification plus subchondral bony defect), which themselves represent a significant damage to the ankle, were treated with MACI/ABG. In addition, we found a high rate of ankle OA on preoperative radiographs in these young patients (stage 1 OA according to the van Dijk classification in eight of the 13 cases). The presence of osteophytes led to the diagnosis of stage 1 OA in all of the cases. OA was present in cases with and without previous operations of the affected OCLT (three of four cases [75%] vs five of nine cases [56%]). Furthermore, OA was also detected before MACI/ABG in both idiopathic and traumatic OCLTs (four of five [80%] vs four of eight [50%]). In other words, OA affected both idiopathic and traumatic cases and also cases without any previous operation of the affected ankle. This finding supports the assumption that OCLTs may lead to the development of diffuse ankle OA. However, osteochondral lesions may also be part of developing OA of the ankle due to other causes. The question is whether the clinical outcome is influenced by the initial OCLT, the surgical intervention, or the potential OA.

There is a lack of evidence about the natural history of OCLTs and the potential association between OCLT and OA in paediatric and adolescent patients. Edmonds et al. reported radiographic outcomes after ankle arthroscopy plus retrograde drilling in 50 ankles after a mean follow-up of 15.1 ± 11.5 months (range 3.2–49.1 months) [20]. The mean patient age at the time of surgery was 13.2 ± 2.7 years. Remarkably, the authors detected worsening signs of OA according to the Kellgren-Lawrence classification in 25% of cases (reported preoperative score: mean 0.42, median 0 vs postoperative score: mean 0.60, median 1). Patient age of 11.5 years was identified as a potential predictive value for advancing Kellgren-Lawrence stage [20]. When comparing our results to the findings of the above mentioned study, it must be considered that the lesion characteristics in both populations were different. Edmonds et al. only included OCLTs with intact overlaying cartilage, whereby the OCLTs within our study all showed significant cartilage damage. Nevertheless, Edmonds et al. also found signs of OA prior to surgical treatment, similar to our study.

We detected van Dijk stage progression on post- compared to preoperative radiographs in only one case. But the differences between post- and preoperative radiographs must be interpreted with caution because the time interval between MACI/ABG and the follow-up radiographs was very different between the cases.

It is unknown whether the natural history of OCLTs differ between children/adolescents and adults. In a 24-year follow-up (range 7–36 years) of 13 paediatric patients with OCLT of the talar dome that were treated conservatively, Wester et al. found abnormalities in five cases in follow-up imaging, such as persistent primary lesion in three cases and loose bodies in two cases [21]. Three of these five patients had mild symptoms at follow-up. The authors concluded that OA is infrequent in paediatric OCLTs [21]. In contrast, Ikuta et al. reported a case of a 12-year-old girl with medial talar OCLT [22]. She was treated conservatively over a period of 10 years. After an interval of 4 years, she developed limitations of recreational activities and signs of OA on follow-up radiographs [22].

Weigelt et al. presented long-term clinical and radiological outcomes of 24 mainly adult OCLT patients that were treated conservatively after a mean follow-up of 14 years (range 11–20 years) [23]. The mean initial patient age was 42 years (range 10–69 years). At follow-up, 29% of the cases showed no signs of OA, 50% stage 1 OA, and 21% stage 2 OA according to the van Dijk classification. In this study, 15 initial radiographs were available that were compared with follow-up radiographs. At the time of follow-up, 11 cases showed no OA progression, and four cases showed progression by one grade. However, the clinical results did not correlate with the radiological findings [23].

In the present study, we did not investigate a potential correlation between the presence of pre- or postoperative OA and OA progression and clinical outcome, since the number of cases was too small to draw reliable conclusions. Furthermore, our cohort represents a highly selected subgroup of OCLT patients with only high-stage OCLTs.

There are several reports of good clinical outcomes following ankle arthroscopy and retrograde drilling [4, 7, 8]. However, this technique is reserved for OCLTs with intact overlaying cartilage [24]. There is a deficit of studies on the treatment of high-stage OCLTs in children and adolescents. We assume that the preoperative OCLT stage (cartilage damage and subchondral bony defect) has a major impact on the long-term clinical and radiological outcome. Several retrospective case series reported heterogeneous clinical and radiographic results of arthroscopic BMS techniques; however, to the best of our knowledge, there is no study investigating the outcome of cartilage replacement therapies (MACI or M-BMS) in combination with ABG or osteochondral transplantation in the paediatric and adolescent population.

Carlson et al. followed 22 OCLT patients with a mean age of 14.4 years (range 8–18 years) with BMS and detected high functional outcomes according to different scores after a mean follow-up of 8.3 years (range 2–27 years) [6]. Remarkably, the authors found van Dijk OA stage 0 in 56%, stage 1 in 38%, and stage 2 in 6% of the cases on postoperative radiographs. Jurina et al. reported the results of arthroscopic BMS in 13 skeletally immature patients with OCLT [5]. They detected good clinical results in 10 and fair clinical results in three cases according to the Berndt and Harty outcome question as well as significant American Orthopaedic Foot and Ankle Society (AOFAS) score improvement compared to the preoperative AOFAS score [5]. Reilingh et al. reported the outcome of operative treatment of skeletally immature OCLT patients with a mean age of 13 years who were treated with fragment fixation (*n* = 9) and debridement/BMS (*n* = 21) [3]. The BMS patients had a good outcome in 13 cases, a fair outcome in three cases, and a poor outcome in five cases, according to the Berndt and Harty outcome question. The median AOFAS score was 95 (range 45–100) at follow-up [3].

In the abovementioned study by Kramer et al. in addition to other outcome measures, the FAOS score was evaluated in 44 patients. The average FAOS score was 77 ± 18, which is comparable to our results (mean FAOS score 78 ± 13) [9]. However, the surgical techniques and lesion characteristics between the study by Kramer et al. and our study were different, which limits the comparability.

The outcomes of BMS techniques decline with increasing defect size and depth [25]. The threshold for cartilage replacement therapies such as MACI or M-BMS is a defect size of 1.0 cm^2^ [10] or 1.5 cm^2^ [12] and greater. Giannini et al. also defined a lesion depth over 5 mm as an indication for bone grafting in chronic OCLTs [12]. M-BMS has the advantage of being a one-step procedure compared to MACI, which requires two operations. M-BMS showed better clinical results compared to microfracture in patellar chondral defects [26], while this was not the case for adult OCLT patients [27].

New therapeutic procedures such as differentiated to chondrocytes bone marrow mesenchymal stem cells cultured on a collagen type I/III scaffold have shown encouraging clinical results in full-thickness cartilage defects of the knee [28]. It remains to be seen whether this technique can also be successfully applied to the ankle joint.

There are recommendations for debridement without BMS for acute and small (< 1cm^2^) or partial thickness OCLTs [12, 25]. A recent review showed good to excellent short- and medium-term outcomes after arthroscopic debridement of focal cartilage lesions of the knee [29].

**Fig. 3:**
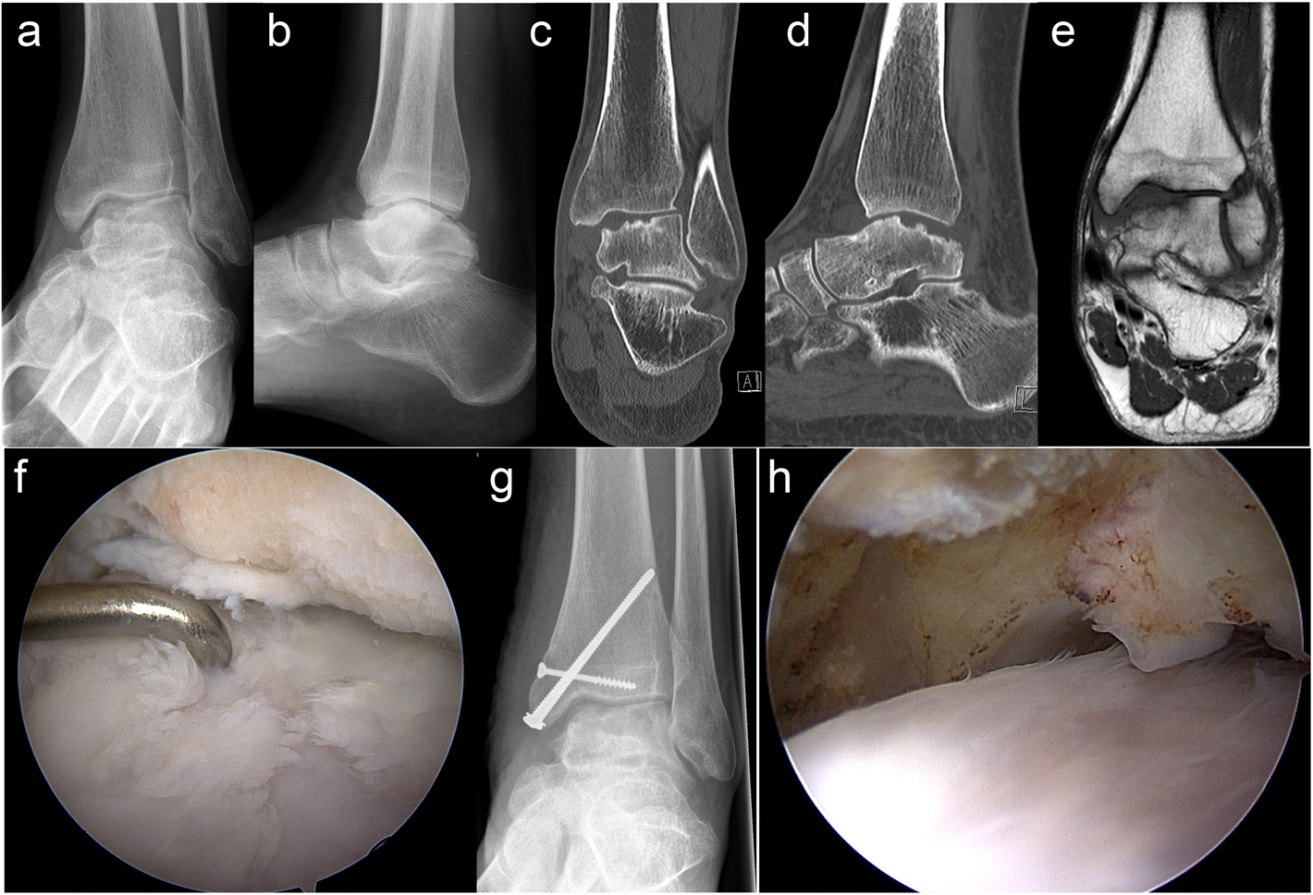
Eighteen-year-old male patient (case 11) with a posttraumatic osteochondral lesion of the medial talus, which was treated with primary combined matrix-associated autologous chondrocyte implantation (MACI) via medial malleolar osteotomy with autologous bone grafting (ABG) from the ipsilateral iliac crest; preoperative antero-posterior (**a**) and lateral (**b**) radiographs; preoperative computed tomography scans in the coronary (**c**) and sagittal (**d**) plane; preoperative magnetic resonance image in the coronary plane (**e**); arthroscopic image at the time of chondrocyte harvesting (**f**) showing the cartilage damage at the medial talus and the status after debridement of a ventral tibial osteophyte; antero-posterior radiograph (**g**) after MACI/ABG showing screw fixation of the medial malleolar osteotomy; arthroscopic image at the time of hardware removal (**h**) 23 months after MACI/ABG showing the cartilage surface at the medial talus

The mean patient age of the present study was higher compared to the abovementioned studies. This is partly because some cases had previous, less invasive surgical interventions at the affected OCLT prior to MACI/ABG, and MACI/ABG was used as a revision technique. Furthermore, MACI/ABG was realised in all cases in this study via a malleolar osteotomy, which should only be performed when the growth plates of the distal tibia/fibula are closed (Fig. 3).

Our results regarding OCLT location are in accord with other findings in paediatric [9] and adult OCLT patients [27, 30], according to which OCLTs occur more frequently at the medial than at the central or lateral talus.

This study has several limitations. First, we could not compare the results of the FAOS and FAAM scores with preoperative baseline values. This is due to the retrospective character of the study. Second, the number of cases is too small to compare different subgroups according to the clinical outcome (e.g., idiopathic vs traumatic). However, we were able to present long-term outcomes of a homogeneous study population after a standardised procedure. To the best of our knowledge, this is the first study reporting PROMs after MACI/ABG in an adolescent population.

Future studies should focus on the long-term outcomes of both operatively and non-operatively treated OCLTs. Because of the rarity of paediatric OCLTs, this should be realised via multicentre registries to generate greater study populations. Different operative techniques could be compared according to different outcome measures. Special attention should be given to the development of OA in the long term and the association between radiographic findings and clinical outcomes.

## Conclusions

PROMs following MACI/ABG for high-stage OCLTs in adolescents are heterogeneous between patients. Long-term limitations seem to especially affect sports and recreational activities. OCLTs are associated with OA, even preoperatively. However, we did not find significant OA progression after MACI/ABG in the long term.

## Data Availability

The datasets generated and/or analysed during the current study are not publicly available due to German and European data protection laws.

## Abbreviations

ABG: Autologous bone grafting
ADL: Activities of daily living
BHL: Berndt-Harty-Loomer
BMI: Body mass index
BMS: Bone marrow stimulation
FAAM: Foot and Ankle Ability Measure
FAOS: Foot and Ankle Outcome Score
FU: Follow-up
ICRS: International Cartilage Repair Society
MACI: Matrix-associated autologous chondrocyte implantation
M-BMS: Matrix-induced bone marrow stimulation
OCLT: Osteochondral lesion of the talus
OA: Osteoarthritis
PROM: Patient-reported outcome measure
QOL: Quality of life
SD: Standard deviation
